# Correction: PINK1 alleviates thermal hypersensitivity in a paclitaxel-induced *Drosophila* model of peripheral neuropathy

**DOI:** 10.1371/journal.pone.0257439

**Published:** 2021-09-10

**Authors:** Young Yeon Kim, Jeong-Hyun Yoon, Jee-Hyun Um, Dae Jin Jeong, Dong Jin Shin, Young Bin Hong, Jong Kuk Kim, Dong Hyun Kim, Changsoo Kim, Chang Geon Chung, Sung Bae Lee, Hyongjong Koh, Jeanho Yun

The affiliation for the eleventh author is incorrect. Sung Bae Lee is not affiliated with #7 but with #6: Department of Brain & Cognitive Sciences, Daegu Gyeongbuk Institute of Science and Technology, Daegu, Republic of Korea.
