# Supplementary material for: PINK1 alleviates thermal hypersensitivity in a paclitaxel-induced Drosophila model of peripheral neuropathy
Source: PLoS One. 2020 Sep 17;15(9):e0239126. doi: 10.1371/journal.pone.0239126 (PMC7498067; doi:10.1371/journal.pone.0239126)
Supplement: S1 Fig — Quantitative analysis of the mitophagy of C4da sensory neurons at abdominal segment A4 in L3 ppk>GFP RNAi and ppk>PINK1 RNAi larvae (n = 5 per group). The results are presented as the mean values, and the error bars represent the SD. Significance was determined by Student’s t-test. NS; not significant. (DOCX) [file pone.0239126.s001.docx]

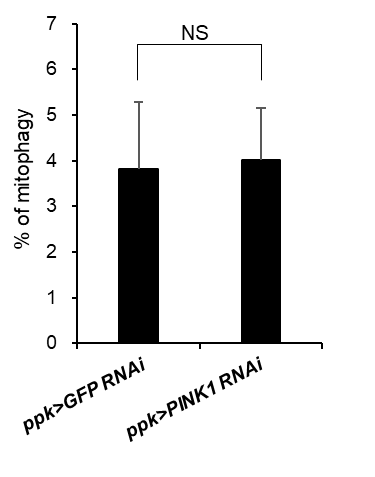


**Figure S1.** Effect of PINK1 knockdown on mitophagy the level in C4da neuron of L3 larvae.

Quantitative analysis of the mitophagy of C4da sensory neurons at abdominal segment A4 in L3 *ppk>GFP RNAi* and *ppk>PINK1 RNAi* larvae (n=5 per group). The results are presented as the mean values, and the error bars represent the SD. Significance was determined by Student’s t-test. NS; not significant.
