# Supplementary material for: PINK1 alleviates thermal hypersensitivity in a paclitaxel-induced Drosophila model of peripheral neuropathy
Source: PLoS One. 2020 Sep 17;15(9):e0239126. doi: 10.1371/journal.pone.0239126 (PMC7498067; doi:10.1371/journal.pone.0239126)
Supplement: Attachment — Submitted filename: Revision letter.docx [file pone.0239126.s002.docx]

March 9, 2020

Dear Editor,

Thank you very much for reviewing our manuscript entitled “PINK1 alleviates thermal hypersensitivity in a paclitaxel-induced *Drosophila* model of peripheral neuropathy” (PONE-D-19-31363). We greatly appreciate the reviewers’ valuable comments and suggestions. We have modified the manuscript extensively and have included additional data based on the reviewers’ suggestions, which we believe have strengthened the manuscript.

We hope that we have fully addressed the concerns of each reviewer and that the revised manuscript now meets the standards for publication in *PLoS One*.

Each concern raised by the reviewers is carefully addressed point-by-point below.

# **Reviewer #1:**

*This study focuses on the role of PINK1 in chemotherapy induced pain. The study is clearly presented and executed reasonably. The results are interesting to both the pain field and probably also the neurodegeneration community.*

*I have only minor comments:*

*1. Please show temperature dose response profiles similar to Fig 1 a for PPK-GFP vs PPK-PINK1 overexpression or control vs RNAi so we understand the baseline difference in nociception.*

**Response:** We appreciate the reviewer for their positive comments. According to the reviewer’s suggestion, we measured the thermal nociceptive response of *ppk>w^1118^* and *ppk>PINK1* L3 larvae to heat probes at different temperatures. We also measured the thermal nociceptive response of *ppk>GFP RNAi* and *ppk>PINK1 RNAi* L3 larvae.

We have added these data as Supplementary Fig. S1 and Fig. S2, respectively, and have modified the Results section accordingly.

*2. PDF figures are of low quality please improve.*

**Response:** The low resolution figures in the PDF file are probably caused by the conversion of the original images to the PDF files. All of the submitted figure images were of 300 dpi resolution. In this revision, we have prepared all the original figure images carefully according to the figure preparation guidelines of PLoS One.

# **Reviewer #2:**

*Kim et al, in this manuscript found that overexpressing pink1 rescue paclitaxel-induced Drosophila model of peripheral neuropathy. This CIPN) model was previously established by other labs. Overall, the phenotype sounds interesting, while the underlying molecular mechanism is elusive. The authors need address the following questions for further consideration.*

*Point 1, data showed in Figure1 is quite similar with a previous publication in DMM PMID: 6031360, where the assay was developed. They just verified this assay and no point to present as a main figure.*

**Response:** As we have described in the Results section, we established a paclitaxel-induced peripheral neuropathy model and phenotype analysis system referring to several recent studies. Although we adopted the paclitaxel treatment paradigm from Zhai’s groups study (Brazil J.M. et al, 2018, Dis Model Mech, doi: 10.1242/dmm.032938), we established the heat probe assay and dendrite structure analysis assays using different system such as different custom-built thermal probes, different fluorescent membrane markers (CD4-tdTomato), and a dendrite analysis system. We believe that verifying the paclitaxel-induced peripheral neuropathy model is an important step for our study and thus, we want keep Figure 1 as a main figure in our manuscript.

*Point 2. Fig2 and Fig4 showed that overexpressing pink1 can rescue branch numbers, dendrite length and relative withdrawal for CIPN, since they addressed the same point, they could be merged in a single figure.*

**Response:** According to the reviewer’s suggestion, we have combined previous Figure 2 and Figure 4 and corrected the manuscript accordingly.

*Point 3. Phenotypical analysis such as branch numbers, dendrite length should also be done for pink1 RNAi in Fig 3. Also, the phenotype when knocking down pink1 in this CIPN model need to be addressed.*

**Response:** According to the reviewer’s suggestion, we have examined the dendrite structure of C4da neurons upon PINK1 knockdown. We found that that both the dendrite length and the number of dendrite branches were not significantly changed upon PINK1 knockdown. These results suggest that knockdown of PINK1 has no effect on the dendrite structure of C4da neurons as well as larval growth.

We have added these data to Figure 3(C, D) and have modified the Results section.

*Point 4. In Fig4, the authors showed that mitophagy defects in this CIPN model can also be restored by overexpressing pink1.*

*This correlation study failed to provide in-depth information about the role of pink1 in this context. A couple of experiments may be helpful to address this question:*

*For instance, is parkin or other autophagic genes involved? Is mitochondrial function normal?*

*Mitochondrial membrane potential or ROS level can be easily tested.*

*In addition, since paclitaxel targets microtube, mitochondrial transport might also be affected. They should test it.*

**Response:** We agree with the reviewer that the analysis of the involvement of Parkin or other autophagy genes would provide valuable information for understanding the role of PINK1. Regrettably, we were not able to perform an additional genetic study because the generation of the *Drosophila* line is not possible within the limited time frame of this revision. Mitochondrial transport was also not examined during this revision due to a lack of a system for the analysis.

During this revision, we further confirmed paclitaxel-induced mitochondrial dysfunction. Because the analysis of C4da neuron-specific changes in mitochondrial membrane potential and mitochondrial ROS is not possible using conventional fluorescence dyes such as TMRM, TMRE, or mitoSOX, we adopted the mitochondrial H_2_O_2_ probe mito-roGFP2-Orp1 (Albrecht, S.C. et al, 2011, Cell Metab, doi: 10.1016/j.cmet.2011.10.010). By expressing mito-roGFP2-Orp1 using a C4da-specific *ppk-GAL4* driver, we were able to analyze mitochondrial ROS changes upon paclitaxel treatment in C4da neurons. We observed that the level of mitochondrial ROS was significantly increased in C4da neurons upon paclitaxel treatment.

We have added these data as Figure 4 and have modified the Results section.

*Point 5. Loss of Pink1/parkin cause neurodegeneration, whether pink1 cause C4da neurons death was not tested in this context.*

**Response:** We understand the reviewer’s concern about the toxic effect of PINK1 in C4da neurons. However, we observed no significant change in larval growth upon either overexpression or knockdown of PINK1. In addition, the dendrite structure of C4da neurons was not significantly changed upon knockdown of PINK1. Thus, these results suggest that the growth and survival of C4da neuron was not affected by PINK1 at least in our experimental setting. We have mentioned this point in the Results section.
