# Supplementary material for: PINK1 alleviates thermal hypersensitivity in a paclitaxel-induced Drosophila model of peripheral neuropathy
Source: PLoS One. 2020 Sep 17;15(9):e0239126. doi: 10.1371/journal.pone.0239126 (PMC7498067; doi:10.1371/journal.pone.0239126)
Supplement: Attachment — Submitted filename: Revision letterR2.docx [file pone.0239126.s003.docx]

May 31, 2020

Dear Editor,

Thank you very much for reviewing our manuscript entitled “PINK1 alleviates thermal hypersensitivity in a paclitaxel-induced *Drosophila* model of peripheral neuropathy” (PONE-D-19-31363R1). We sincerely appreciate the reviewers’ valuable comments and suggestions. We have modified the manuscript extensively and have included additional data based on the reviewers’ suggestions, which we believe have strengthened the manuscript.

We hope that we have fully addressed the concerns of each reviewer, and that the revised manuscript now meets the standards for publication in *PLoS One*.

Each concern raised by the reviewers is carefully addressed point by point below.

*Reviewer #1: I think this manuscript is almost finished, but a few issues need to be addressed.*

*1. I think it is essential to show the raw control vs pink1 (overexpression or RNAi) withdrawal responses side by side with significance assessed to highlight if there is a significant baseline difference in the main figures of the manuscript, then show the relative changes from there. The issue is the baseline looks different, and the response to paclitaxel is different, and both messages should be clearly presented in the main figures to help the reader fully understand the results.*

**Response:** We agree with the reviewer that we should show the raw thermal nociceptive response in the main figure before we show the relative withdrawal response. According to the reviewer’s suggestion, we now show the thermal nociceptive responses of control (*ppk>w^1118^*) and PINK1-overexpressing (*ppk>PINK1*) larvae in Fig. 2A. The thermal nociceptive responses of control (*ppk>GFP RNAi*) and PINK1 RNAi (*ppk>PINK1 RNAi*) larvae are also shown in Fig. 3A.

In addition, we show the raw heat probe assay results of control (*ppk>w1118*) and PINK1-overexpressing (*ppk>PINK1*) larvae in Fig. 2B, and the relative withdrawal changes are shown in Fig. 2C.

We appreciate the reviewer for pointing out this issue. By considering the effects of PINK1 overexpression and knockdown on thermal nociception and the dendrite structure of C4da neurons, we found that PINK1 may alleviate paclitaxel-induced thermal hypersensitivity by means other than preventing alterations in the sensory dendrites of C4da neurons. Thus, we modified the manuscript as we discuss in our next response below.

*2. Figure 2a as presented makes it look like there is no baseline change in nociceptive response to 40C, but figure S1 40C shows what looks like a strong difference at 40C, and I feel this needs to be dealt with upfront so that the data and normalized differences don’t get misinterpreted. Similar issue with Figure 2a vs S2. Then for both the authors should discuss how the baseline difference could confound interpretation of the paclitaxel data so the reader is led toward a more complete understanding of the results.*

**Response:** We agree with the reviewer that the effect of PINK1 overexpression should be considered carefully. By analyzing the results upon PINK1 overexpression, we found that PINK1 expression significantly changed thermal nociception as well as the dendrite structure of C4da neurons. Nevertheless, our results suggest that PINK1 expression significantly alleviates paclitaxel-induced thermal hypersensitivity in larvae, and that PINK1 may regulate thermal nociception and dendrite structure through different mechanisms. We believe that the results obtained from PINK1-knockdown experiments further support this notion. Therefore, we have carefully revised the manuscript to clearly state our interpretation of the results. The major changes are shown below.

(Page 9 line 1- Page 10 line 3)

**Ectopic expression of PINK1 rescues the thermal hypersensitivity phenotype induced by paclitaxel treatment**

To examine the effect of PINK1 on paclitaxel-induced thermal sensory hypersensitivity, we expressed PINK1 specifically within C4da neurons using the *ppk*-*GAL4* driver [20] and examined thermal nociception upon paclitaxel treatment. We found that PINK1 overexpression significantly reduced thermal sensitivity (Fig. 2A). Larvae expressing PINK1 exhibited approximately 13% to 48 % increased withdrawal latency with different heat probe temperature, suggesting that PINK1 is implicated in thermal nociception. Interestingly, we observed that PINK1 overexpression within C4da neurons significantly suppressed the sensitivity to heat upon paclitaxel treatment. While paclitaxel reduced the MWL by 2.41 sec (from 5.88 sec to 3.47 sec) in control larvae (*ppk>w^1118^*), the MWL was decreased by 0.99 sec (from 8.08 sec to 7.09 sec) upon paclitaxel treatment in larvae expressing PINK1 (*ppk>PINK1*) (Fig. 2B). In terms of the relative changes, paclitaxel reduced the MWL by 42% in control larvae, while the MWL was decreased by only 12% in larvae expressing PINK1 upon paclitaxel treatment (Fig. 2C). These results suggest that PINK1 expression significantly alleviated paclitaxel-induced thermal hypersensitivity in a thermal nociception assay.

We then assessed the dendritic arborization of C4da neurons, which is known to be associated with the thermal nociception [8]. We observed significant decreases in dendrite length and the number of branch points in larvae expressing PINK1 (Fig. 2D and E). Ectopic expression of PINK1 reduced the dendrite length and the number of branch points of C4da neurons by 51% and 53%, respectively, compared to control larvae, suggesting that the reduced thermal sensitivity upon PINK1 expression may be due to decreased dendritic arborization of C4da neurons. Because PINK1 expression suppressed paclitaxel-induced thermal hypersensitivity, we expected that dendritic arborization would increase less in larvae expressing PINK1 than in control larvae. However, paclitaxel treatment further increased dendritic arborization in larvae expressing PINK1 compared with control larvae (Fig. 2D and E). Quantitative analysis of multiple C4da neurons (n≧ 5) revealed that the dendrite length was increased upon paclitaxel treatment by 32% in control larvae and by 42% in larvae expressing PINK1 (Fig. 2E). The number of dendrite branch points was increased upon paclitaxel treatment by 49% and 70% in control larvae and in larvae expressing PINK1 respectively (Fig. 2E). These results suggest that suppressing alteration of dendrite arborization may not be the only mechanism through which PINK1 alleviates the thermal hypersensitivity induced by paclitaxel treatment.

(Page 10 line 13- Page 11 line 5)

**PINK1 knockdown induces thermal hypersensitivity.**

To further test the role of PINK1 in thermal nociception, we next examined the effect of specific knockdown of PINK1 expression in C4da neurons. Whereas ectopic PINK1 expression reduced thermal sensitivity in the heat probe assay, C4da neuron-specific knockdown of PINK1 using the *ppk-GAL4* driver resulted in significant sensitization of L3 larvae to different heat probe temperatures in the thermal nociception assay (Fig. 3A). PINK1 knockdown decreased the withdrawal latency to the 40 ºC heat probe to 41% of the control level (Fig. 3B), confirming that PINK1 plays an important role in thermal nociception.

To understand the role of PINK1 in the sensory dendrites of C4da neurons, we also examined the dendritic structure of C4da neurons upon C4da neuron-specific knockdown of PINK1. Interestingly, we observed no change in the dendritic structure of C4da neurons upon PINK1 knockdown (Fig. 3C). The quantitative analysis of multiple C4da neurons (n≧ 5) also showed that both dendrite length and the number of dendrite branches (Fig. 3D) were not significantly changed upon PINK1 knockdown. These results indicate that knockdown of PINK1 in C4 da neurons induces an increase in thermal nociception without changes in the dendritic structure. We also found that the size of larvae was not significantly changed upon PINK1 knockdown (Fig. 3E), confirming that PINK1 has no effect on larval growth. These results suggest that PINK1 may separately regulate thermal nociception and dendrite structure through independent mechanisms.

*3. The figures are out of order in the PDF for some reason, also the figure quality is still poor for some reason, please make sure the final published figures are legible, I understand the PDF conversion process can cause this.*

**Response:** We apologize for this error. We carefully checked the order of the figures this revision.

Regarding the quality of the figures, we have prepared all the original images carefully according to the figure preparation guidelines of *PLoS One*. All of the submitted figure images are of 300 dpi resolution. Thus, we believe that the quality of original figures will meet the standard of the journal.

*4. Figure 1C etc, “relative withdrawal (%)” is sort of confusing, as presented it at first glance seems like paclitaxel reduces sensitivity. It might make more sense to present the data as % sensitization or similar. In general I find “decreased MWL” difficult to conceptualize and I feel “sensitization” is easier for the non-pain expert to understand.*

**Response:** To address the reviewer’s concern about the term “relative withdrawal (%)”, we instead use the term “withdrawal latency (% baseline)”, which is commonly used in pain experiments, in Fig. 2C. The results in Fig. 1C are now expressed as “withdrawal latency” instead of the relative values.

To facilitate readers’ understanding, we have reduced the use of the term “mean withdrawal latency (MWL)” and revised the manuscript by using the term “thermal sensitivity” or “sensitization” as shown below.

(Page 9, line 5)

We found that PINK1 overexpression significantly reduced thermal sensitivity (Fig. 2A). Larvae expressing PINK1 exhibited approximately 13% to 48% increased withdrawal latency with different heat probe temperatures,…

(Page 10, line 15)

Whereas ectopic PINK1 expression reduced thermal sensitivity in the heat probe assay, C4da neuron-specific knockdown of PINK1 using the *ppk-GAL4* driver resulted in significant sensitization of L3 larvae to different heat probe temperatures in the thermal nociception assay (Fig. 3A). PINK1 knockdown decreased the withdrawal latency to the 40 ºC heat probe to 41% of the control level (Fig. 3B),…

*Reviewer #2: The authors have addressed all the concerns I have. It would be great if they had some mechanistic analysis rahter than the descriptive data only.*

**Response:** We agree with the reviewer that understanding the precise mechanism through which PINK1 expression suppresses paclitaxel-induced thermal hypersensitivity is important. In particular, how PINK1 reduces paclitaxel-induced mitochondrial dysfunction in sensory neurons needs to be explored in further studies. We are currently conducting experiments to understand the molecular mechanisms and hope that we are able to report the results soon.
