# Supplementary material for: PINK1 alleviates thermal hypersensitivity in a paclitaxel-induced Drosophila model of peripheral neuropathy
Source: PLoS One. 2020 Sep 17;15(9):e0239126. doi: 10.1371/journal.pone.0239126 (PMC7498067; doi:10.1371/journal.pone.0239126)
Supplement: Attachment — Submitted filename: Revision letterR3.docx [file pone.0239126.s004.docx]

August 7, 2020

Dear Editor,

Thank you very much for reviewing our manuscript entitled “PINK1 alleviates thermal hypersensitivity in a paclitaxel-induced *Drosophila* model of peripheral neuropathy” (PONE-D-19-31363R2). We sincerely appreciate the reviewers’ valuable comments and suggestions. We have modified the manuscript and have included additional data based on the reviewers’ suggestions, which we believe have strengthened the manuscript.

We hope that we have fully addressed the concerns of each reviewer, and that the revised manuscript now meets the standards for publication in *PLoS One*.

Each concern raised by the reviewers is carefully addressed point by point below.

Reviewer #1: The authors have addressed all of my concerns and I believe the manuscript is suitable for publication.

Reviewer #2: The current manuscript is significantly improved, however, it is overall descriptive, mechanistic analysis is largely missing.

In Figure 5, the authors observed that paclitaxel induced mitophagy was suppressed by pink1 OE using mitoKeima reporter. The authors then claimed that PINK1 reduced paclitaxel-induced increases in mitophagy levels.

Whether pink1 suppress or accelerate mitophagy in this context needs to be clarified, since previously reports showed that pink1/parkin KD muscles or DA neurons have less mitophagy activity in Drosophila (eLife 2018;7:e35878 doi: 10.7554/eLife.35878).

The authors can easily address this by manipulating downstream events of mitophagy, such as lysosome and proteasome activity in pink1 LOF background.

**Response:** We understand the reviewer’s concern about the mitophagy change upon PINK1 overexpression. We repeated the Fig. 5 experiment and mitophagy analysis with larger number of samples and found that the basal mitophagy level of the C4da neuron was not significantly changed upon PINK1 expression. As the reviewer mentioned, Wim Vandenberghe’s group has shown that loss of PINK1 and knockdown of parkin abolish the age-dependent increase of mitophagy in muscle while basal mitophagy is unchanged (Cornelissen T et al, 2018, eLife, 7:e35878). We have also previously shown that the mitophagy level of DA neurons was not changed by knockdown of PINK1, but mitophagy inductions upon hypoxia, and rotenone treatment were abolished (Kim YY et al, 2019, FASEB J, 33:9742-9751. doi: 10.1096/fj.201900073R), suggesting that PINK1 function is dispensable for basal mitophagy. We also observed that the mitophagy level of C4da neurons was not changed by knockdown of PINK1. Given the significant increase in thermal sensitivity upon PINK1 knockdown, the unchanged mitophagy activity suggested that PINK1 may restore mitochondrial homeostasis through mechanisms other than mitophagy. Previous studies have indicated that PINK1 controls mitochondrial quality through additional methods such as regulating complex I activity and mitochondrial transport (Reviewed in Voigt A et al, 2016, J Neurochem, 139:232-239, doi: 10.1111/jnc.13655; Steer EK et al, 2015, Antioxid Redox Signal, 22(12):1047-1059 doi: 10.1089/ars.2014.6206). A body of studies even suggest that PINK1 suppresses mitophagy activity in certain cellular contexts through a direct or indirect manner (Reviewed in Steer EK et al, 2015, Antioxid Redox Signal, 22(12):1047-1059 doi: 10.1089/ars.2014.6206). Therefore, the exact mechanism by which PINK1 ameliorates paclitaxel-induced mitochondrial dysfunction in C4da neuron should be carefully determined in further studies.

We have revised the discussion to clearly state these points.

We replaced Fig. 5B with a new results and added the mitophagy results upon PINK1 knockdown as Supplementary FigS1.
